# Temperature Dependence of the Hall and Longitudinal Resistances in a Quantum Hall Resistance Standard

**DOI:** 10.6028/jres.110.078

**Published:** 2005-10-01

**Authors:** J. Matthews, M. E. Cage

**Affiliations:** University of Maryland, College Park, MD 20742; National Institute of Standards and Technology, Gaithersburg, MD 20899-8172

**Keywords:** quantum Hall effect, resistance standard, temperature dependence

## Abstract

We present detailed measurements of the temperature dependence of the Hall and longitudinal resistances on a quantum Hall device [(GaAs(7)] which has been used as a resistance standard at NIST. We find a simple power law relationship between the change in Hall resistance and the longitudinal resistance as the temperature is varied between 1.4 K and 36 K. This power law holds over seven orders of magnitude change in the Hall resistance. We fit the temperature dependence above about 4 K to thermal activation, and extract the energy gap and the effective g-factor.

## 1. Introduction

The quantum Hall effect was discovered by von Klitzing, Dorda, and Pepper in 1980 [1]. They reported that measurements of the Hall resistance *R*_H_ on Si MOSFETS showed a step-like structure at high magnetic fields. The plateaus of these steps were found to be quantized to values very close to *h*/*ie*^2^, where *h* is the Planck constant, *e* is the electron charge, and *i* is an integer. Von Klitzing et al. recognized immediately the implications of this result for resistance metrology. Soon after the discovery of the quantum Hall effect in Si MOSFETS, it was observed in other devices containing two-dimensional electron gases (2DEGs), such as GaAs/AlGaAs heterostructures [2], and InGaAs/InP devices [3].

While the quantization was shown to be independent of the material properties of the sample [4–6], and the precision of quantum Hall measurements continually improved, it was found that finite temperature and current could both lead to deviations from the zero temperature value of the Hall resistance. Several investigations [3,7–18] measured the temperature dependence of the Hall resistance across the device, and the longitudinal resistance *R_x_* along the device, over the following years. However, few experiments could achieve a precision greater than 10^−7^ *h*/*e*^2^, including metrology laboratories.

It is clear that since any measurement must necessarily be made at finite temperature, understanding the temperature dependence of the quantum Hall resistance is critical if we wish to establish a resistance standard based on this value. In practice, one can typically cool the sample to temperatures where no temperature dependence is measurable to the precision of the measurement. However, in order to establish whether or not the temperature is “cold enough” requires an understanding of the physical origin of the temperature dependence. Only then can we be confident that we are approaching the zero-temperature limit.

In this article, we present the results of a set of experiments investigating the temperature dependence of the device, known as GaAs(7), in the integer quantum Hall regime. GaAs(7) is one of the GaAs/AlGaAs heterostructures used at the National Institute of Standards and Technology (NIST) to maintain the unit of resistance for the United States of America.

Although remarkable progress has been made [19–22], a complete theory of the quantum Hall effect is still missing. Nonetheless, there have been serious attempts to explain the physics behind many of the key features of the quantum Hall effect. Plausible explanations for the temperature dependence of the Hall and longitudinal resistances have been proposed for different temperature regimes. In particular, at high temperatures (typically several kelvin) thermal activation across an energy gap generally explains the experimental data satisfactorily. At lower temperatures, where thermal activation is “frozen out,” a form of variable range hopping devised by Efros and Shklovskii [23, 24] is often used to explain the data. However, early results on GaAs(7) and other devices by Cage et al. [13] did not fit either of these models.

There have been a multitude of experiments performed on the quantum Hall effect; in particular, a number of experiments measuring temperature dependences of transport properties [3,7–18]. However, whether due to the nature of the devices, limitations of the measurement systems, or because the experiment was examining some other aspect of the quantum Hall effect, a limited number of results have been reported which have a direct bearing on the regime of interest for resistance metrology [13,17,18].

The experiments described in this article were carried out with the primary goal of exploring the temperature dependence of the quantum Hall resistance on a standards-quality device. Essentially two kinds of experiments were performed. In the first method the Hall and longitudinal voltages were read directly with 8 1/2 digit digital voltmeters (DVMs) while the magnetic field was swept from 0 T to 13.3 T at constant current and constant temperature. This method is the most efficient and flexible way of accumulating data, but the accuracy is limited to about 10^−5^ *h*/*e*^2^. Measurements were made using this method over the temperature range 1.4 K to 34 K. The second method made use of specialized systems to measure either the Hall resistance or the longitudinal resistance at fixed magnetic field, and at constant current and temperature. These measurement systems can be used only close to the Hall plateau centers, where the Hall resistance *R*_H_ approaches its nominal value, and the longitudinal resistance *R_x_* approaches zero. The uncertainties for this method were typically about 10^−8^ *h*/*e*^2^ for the Hall resistance, and 10^−9^ *h*/*e*^2^ for the longitudinal resistance. Measurements at *i* = 4 were made between 1.4 K and 4.2 K using this second method, and measurements at *i* = 2 were made between 1.4 K and 7.0 K.

## 2. Measurement System

**Fig. 1:**
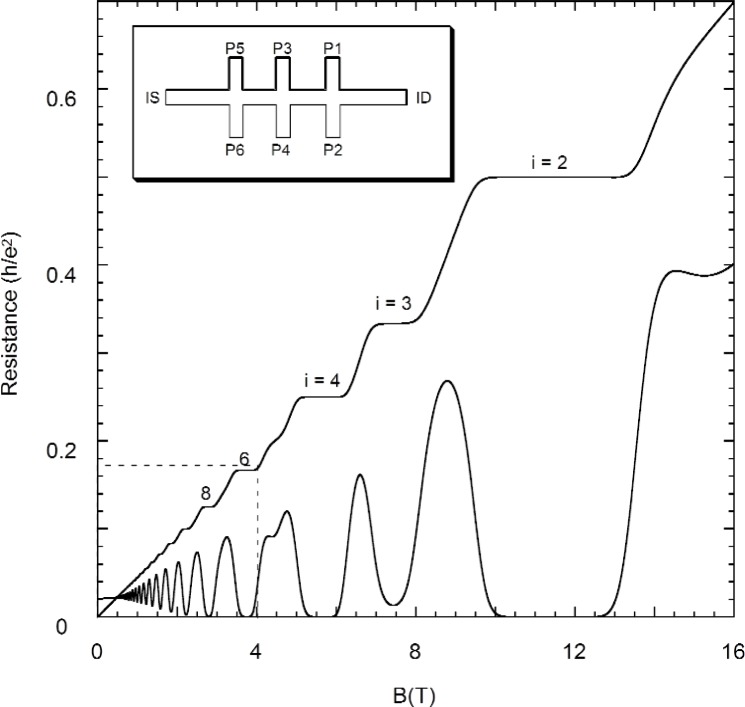
Hall (*R*_H_) and longitudinal (*R_x_*) resistances of GaAs(7) in units of *h*/*e*^2^ as a function of magnetic flux density *B*. Representative plateau numbers are labeled. The top left inset shows a schematic of the device with the probe sets labeled. *R*_H_ was measured across P3–P4. *R_x_* was measured across P2–P6. The dc bias current is connected across IS-ID. The region enclosed by the dashed line is shown magnified in Fig. 2(a).

The quantum Hall measurements were made on a single GaAs/AlGaAs heterostructure which we refer to from here on as “GaAs(7).” GaAs(7) is a GaAs/Al*_x_*Ga_1−_*_x_*As (*x* = 0.31) device produced at Bell Laboratories in the early 1980s by molecular beam epitaxy. The total length of the device is about 5.5 mm. The width of the Hall bar is 0.4 mm, and the separation between neighboring Hall probes is 1.0 mm. A schematic diagram of the device is shown in the inset of Fig. 1. It has an electron number density *n*_s_ = 5.2 × 10^15^ m^−2^, and a zero-field mobility *μ*_s_ = 11.1 m^2^/Vs.

The device was mounted on a sample holder within a variable temperature insert (VTI) at the center of a superconducting magnet, with a maximum applied magnetic flux density of 16 T. The device was immersed in liquid 4He at a temperature of 4.2 K, cooled to 1.4 K by vacuum pumping, and raised above 4.2 K by using a heater and temperature controller. Two calibrated Cernox^1^ thermometers were located close to the sample. One, just below the sample, was mounted on the VTI. The second, just above the sample, was mounted on the sample probe. Having two thermometers bracketing the sample improved temperature control, since it allowed for an estimate of thermal gradients in the neighborhood of the device. The estimated uncertainty in the temperature due to temperature control, thermal gradients, and uncertainties in the thermometer calibrations, was about 10 mK below 4.2 K. At higher temperatures the uncertainty increased because the temperature became harder to control, and thermal gradients increased.

In order to improve the signal-to-noise ratio, and because the signals were frequently very small, all electrical components of the experiment were double-shielded. For components such as switch boxes or patch panels, this involved using two nested layers of electrically insulated aluminum boxes to enclose all electrical connections. All leakage resistances were checked to be at least 10^13^ Ω.

There were two distinct methods for making voltage measurements on the device. The simplest, which for many purposes was sufficient, was to measure the voltage across the device directly with one or more digital voltmeters (DVMs). The device was connected in series with a temperature-controlled, wire-wound resistor, with a value trimmed to within about 1 × 10^−6^ of the Hall resistance *R*_H_ at *i* = 4 (i.e., 6 453.201 75 Ω). The drift in this resistor has been documented at about 5 × 10^−8^ *R*_H_/year [25]. The voltage across the resistor was measured to determine the current *I* flowing through the device. *R*_H_ = *V*_H_/*I* and *R_x_* = *V_x_*/*I*, where *V*_H_ and *V_x_* are the voltages measured across the device, and along the device, respectively. The magnet current was used to determine the magnetic field at the device.

The second method was used whenever greater precision was required at the centers of Hall plateaus or *R_x_* minima. First, in order to reduce the random uncertainty due to noise, measurements were made at constant magnetic field, current and temperature, and continuously averaged until the desired uncertainty level was reached. Second, a system of switching was included in the measurement to reduce the effect of certain systematic effects, such as thermal EMFs, current drift, asymmetrical leakage resistances, etc. A specialized custom-built measurement system POTSYS was used (Ref. [26]), with low noise current sources made from mercury batteries, a nanovoltmeter to amplify small signals, and mechanical rotary switches. Power to the switches was turned off during measurements to reduce electrical noise. The experimental setup and procedure is described in more detail in Ref. [27].

A typical magnetic field sweep is shown in Fig. 1. The Hall (*V*_H_) and longitudinal (*V_x_*) voltages were measured at a constant current of 25 μA, as the magnetic field was swept up from 0 T to 16 T. The temperature, which was the base temperature of the VTI, was 1.4 K. The entire field sweep took about 1 hour to complete.

**Fig. 2:**
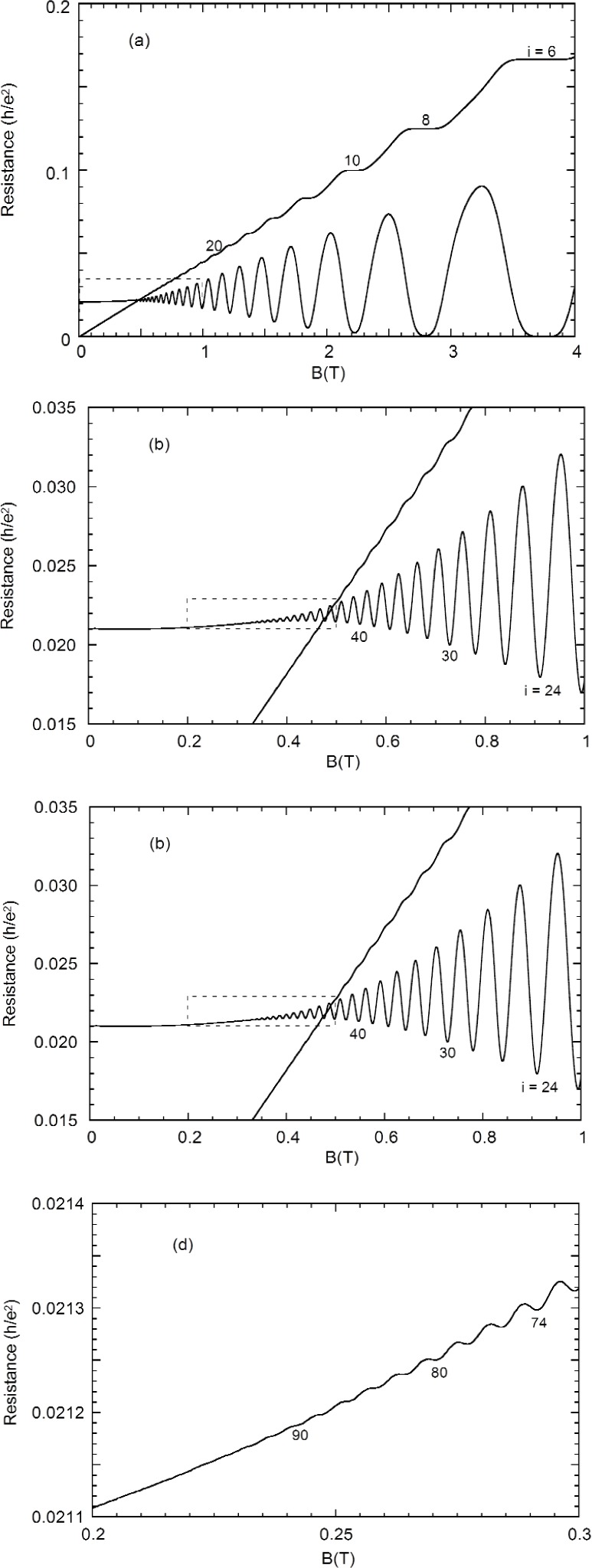
The Hall (*R*_H_) and longitudinal resistances (*R_x_*) of GaAs(7) as a function of magnetic field. The insets of (a), (b), and (c) are magnified in (b), (c), and (d), respectively. Representative quantum numbers are labeled. Note that we can resolve *R_x_* minima to at least *i* = 90.

Except for the *i* = 3 plateau, no other odd integer plateaus are visible at this temperature. To highlight the detail observable in a single sweep, successively magnified views are shown in Fig. 2. Figures 2(c) and 2(d) were obtained under the same conditions, but at a significantly slower sweep rate, to ensure smooth curves. Notice that indentations in the *R_x_* curve can be identified with filling factors of over *i* = 90.

## 3. Temperature Dependence of *R_x_*

**Fig. 3:**
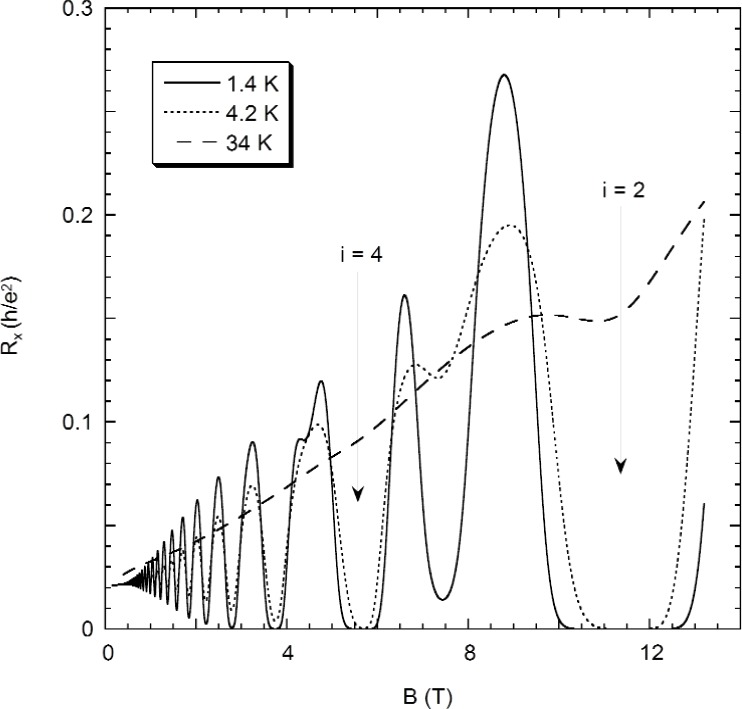
The longitudinal resistance *R_x_* in units of *h/e*^2^ at 1.4 K (solid line), 4.2 K (dotted line), and 34 K (dashed line).

**Fig. 4:**
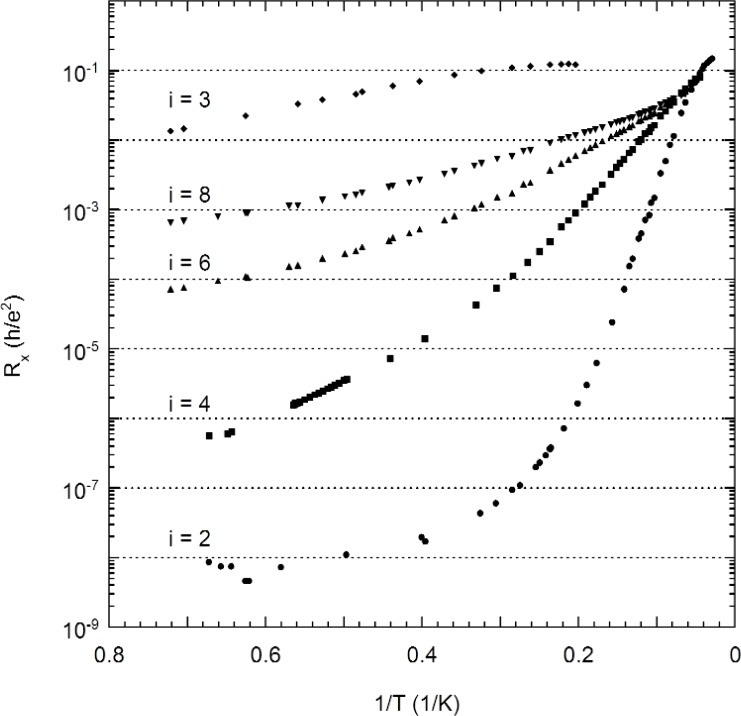
Arrhenius plot of the temperature dependence of *R_x_* for *i* = 2 (circles), *i* = 4 (squares), *i* = 6 (upward triangles), *i* = 8 (downward triangles), and *i* = 3 (diamonds).

Three magnetic field sweeps of *R_x_* at 1.4 K, 4.2 K, and 34 K are shown in Fig. 3, using probe set P2–P6. We note that at 34 K only the *i* = 2 minimum is still discernable. By analyzing a number of sweeps such as these, combined with data taken at constant field and current by using the precision measurement system POTSYS [26], we determined the temperature dependence of *R_x_* at the five most significant *R_x_* minima (*i* = 2, 3, 4, 6, 8), as shown in Fig. 4.

We note here that we were able to measure *R_x_* over more than seven orders of magnitude. There are essentially two temperature ranges of interest. Above about 4 K the dominant conduction mechanism is thermal activation. Here temperatures are high enough that an electron can be thermally excited across the cyclotron energy gap into the mobility edge,

$$R_{x}\left(T\right)=R_{0}e^{-\Delta E/2kT}.$$ (1)

Thermal activation is best viewed on an Arrhenius plot, which shows ln *R_x_* as a function of inverse temperature. Figure 4 shows such an Arrhenius plot of the *R_x_*(*T*) data for filling factors *i* = 2, 4, 6, 8 and 3. The deviation from activation above 12 K is likely due to the rapidly changing electron number density above this temperature. It is possible that the high temperature can excite electrons into the second subband of the 2DEG, breaking the two-dimensional nature of the system. Because of this, the fits to thermal activation were restricted to temperatures below 12 K.

At lower temperatures (below about 4 K) the contribution to the conductivity from thermal activation decreases sharply, and other conduction processes take over. It is commonly believed that variable range hopping (VRH) describes transport in this regime. In VRH theory [28] finite overlap of the wavefunctions of the localized states allows electrons to tunnel between these states. In the presence of an electric field, this tunneling is sufficient to generate a current. One commonly accepted result, due to Efros and Shklovskii [23, 24], is

$$R_{x}=\frac{a}{T}e^{\sqrt{T_{0}/T}}$$ (2)

where *T*_0_ is related to the localization length *ξ* by

$$kT_{0}\left(v\right)=C\frac{e^{2}}{4\pi \varepsilon_{r}\varepsilon_{0}\xi \left(v\right)}$$ (3)

where *v* is the filling factor, which becomes *i* at integer values. *C* ≅ 6.2 in two dimensions, and the relative permittivity *ε*_r_ ≅ 13 for GaAs.

**Fig. 5:**
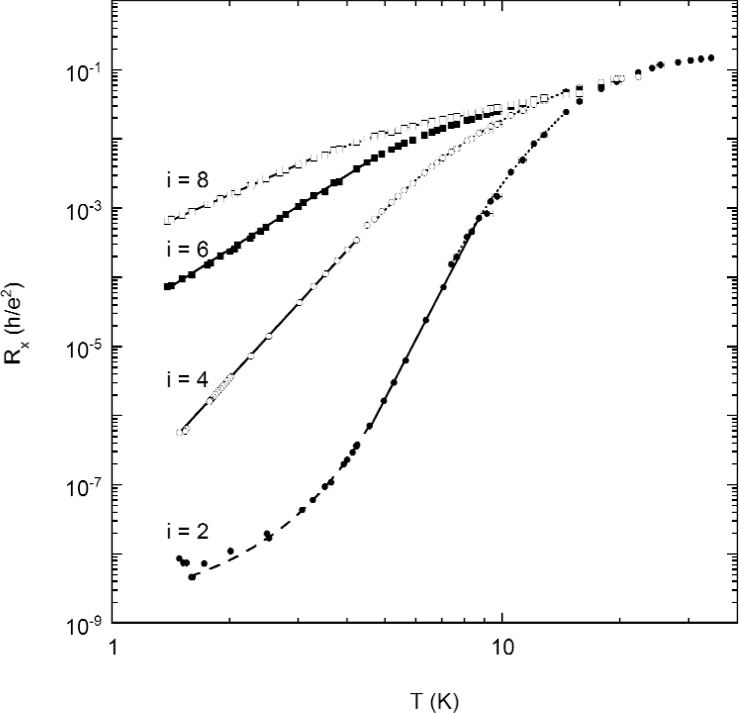
Temperature dependence of *R_x_* for *i* = 2 (solid circles), *i* = 4 (open circles), *i* = 6 (solid squares), and *i* = 8 (open squares). Note the power law dependence at low temperature for *i* = 4, 6, and 8. The solid lines are power law fits. The dotted lines are fits to thermal activation. The dashed line is a fit to exponential-like behavior, as described in the text.

Figure 5 shows the same *R_x_*(*T*) data as in Fig. 4, but recast as a log-log plot. Notice that the low temperature points (with the exception of *i* = 2) fall very naturally on a straight line, hinting at a power law dependence on temperature. On a log-log scale, any of the variable range hopping theories would predict some bending of the data to the right of the straight line at low temperatures. There is no evidence for this at all. In fact, for the *i* = 2 minimum the data bends to the left.

Also shown in Fig. 5 are least squares fits to *R_x_*(*T*). The dotted lines are fits to thermal activation {*R_x_* = *R_x_*_0_exp[(−(*T*_0_/*T*)]}. The solid lines are fits to power laws (*R_x_* = *aT^γ^*), The dashed lines are fits to the empirical fit *R_x_* = *R_x_*_2_exp(*T*/*T*_2_)*^α^* for low temperature values of the *i* = 2 plot. The parameters from the fits are shown in Table 1. Note the dramatic temperature dependence in the power law (solid line) region of *i* = 2 and *i* = 4 which vary as *T*^10.9^ and *T* ^6.1^, respectively. This would make an exceptionally sensitive thermometer over the temperature range 4 K to 8 K for *i* = 2, and 2 K to 7 K for *i* = 4. The temperature dependences are much less dramatic in the power law region: *T* ^3.6^ and *T* ^6.1^, respectively

## 4. Relationship Between *R*_H_ and *Rx*

In this section we discuss the relationship between *R_x_* and ∆*R*_H_, which is the deviation of *R*_H_ from *h/ie*^2^. This is perhaps one of the most important results from a metrological perspective, since frequently one uses *R_x_* as a guideline for identifying proximity to *h/ie*^2^ in the Hall resistance.

**Fig. 6:**
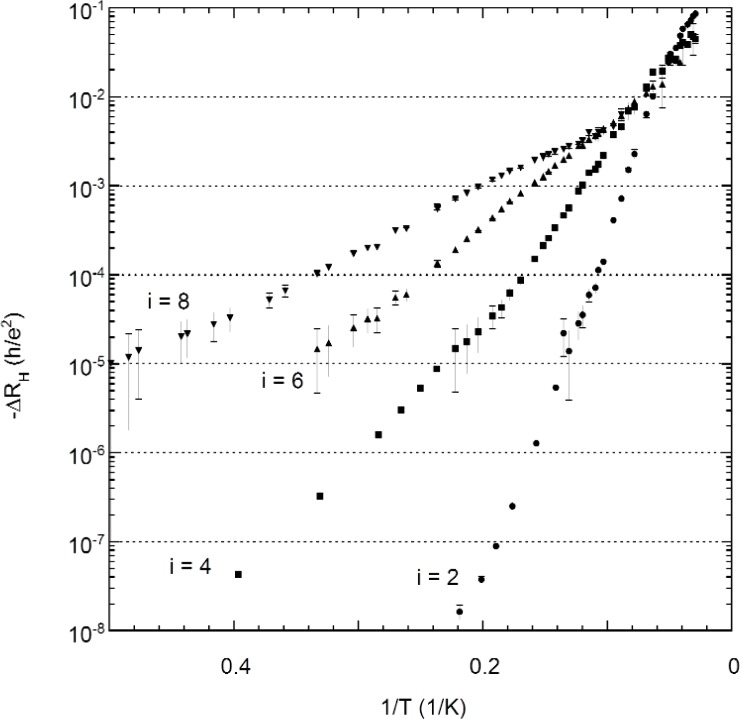
Arrhenius plot of the temperature dependence of the deviation of the Hall resistance from *h/ie*^2^ for *i* = 2 (circles), *i* = 4 (squares), *i* = 6 (upward triangles), and *i* = 8 (downward triangles). The uncertainty in the measurements, as indicated by the error bars, was significantly reduced for *i* = 2 and *i* = 4 by using a precision measurement system.

To motivate this discussion, let us first examine the temperature dependence of *R*_H_ at the *R_x_* minima. Figure 6 shows a plot of ∆*R*_H_ for probe set P3–P4 as a function of temperature on a log-log scale. While no activation or variable range hopping fits are shown, it is clear from the figure that the temperature dependence of *R*_H_ bears at least a qualitative resemblance to that of *R_x_* in Fig. 4. It appears to follow a power law at low temperatures, and possibly activation at higher temperatures.

*R*_H_ is a more difficult quantity to measure than *R_x_*, hence the larger error bars in the figure. The highest precision and accuracy for the *R*_H_ measurements was about 10^−8^ *h/e*^2^ when using the potentiometric measurement system POTSYS.

Figure 7 shows a log-log plot of − ∆*R*_H_ for probe sets P1–P2, P3–P4, and P5–P6 against the *R_x_* P2–P6 probe set. The digital voltmeter portion of the data was obtained by measuring − ∆*R*_H_ and *R_x_* simultaneously at different temperatures. For the POTSYS portion of the data − ∆*R*_H_ and *R_x_* were measured sequentially for each temperature. The most striking feature is that all three Hall probe sets appear to follow a power law over the entire temperature range, including that above 10 K. The straight lines are weighted least squares fits to a power law, − ∆*R*_H_ = s*R*^δ^*x*. The parameters of the fit are given in Table 2.

For *i* = 6 and *i* = 8 the data were obtained directly from the DVMs, which explains why the resolution is much lower than for the *i* = 2 and *i* = 4 plots, which incorporate data obtained using POTSYS.

All the plots show strong evidence for power law dependence over the entire temperature range, and with the exception of *i* = 4, there is very little probe set dependence. The exponent of the power laws increase with increasing filling factor, averaging 1.25 for *i* = 2, 1.44 for *i* = 4, 1.75 for *i* = 6, and 1.97 for *i* = 8.

However, *i* = 4 stands out, since it shows a much more distinctive probe set dependence than any other filling factor. Curiously, this probe set dependence is evident only at low temperatures; specifically, between 1.4 K and 4 K, which is where we observed a power law dependence on temperature for *R_x_*, as shown in Fig. 5. At higher temperatures than this all three probe sets converge to the fit to the P3–P4 probe set.

Note that, as can be seen from Fig. 7(a), the relationship $-\Delta R_{H}=\;0.82{R_{x}}^{1.25}$ for *i* = 2 holds over at least seven orders of magnitude in − ∆*R*_H_

Since we know the temperature dependence of *R_x_*, and now we know the relationship between *R_x_* and ∆*R*_H_, we can determine the temperature dependence of ∆*R*_H_. When thermal activation is observed in both *R_x_*(*T*) and ∆*R*_H_(*T*),

$$R_{x}\left(T\right)=R_{x0}\exp\left(-\Delta E_{x}/2kT\right)$$ (4)

$$\Delta R_{H}\left(T\right)=\Delta R_{H0}\exp\left(-\Delta E_{H}/2kT\right).$$ (5)

Eliminating *T* from Eqs. (4) and (5), we obtain the following relationship between *R_x_*(*T*) and ∆*R*_H_(*T*),

$$\Delta R_{H}\left(T\right)=\Delta R_{H0}{\left(\frac{R_{x}\left(T\right)}{R_{x0}}\right)}^{\Delta E_{H}/\Delta E_{x}}$$ (6)

if ∆*E_x_* and ∆*E*_H_ are *different* energy gaps for *R_x_* and ∆*R*_H_, respectively. From Eq. (6) we can see that −∆*R*_H_, follows a power law dependence on *R_x_*, where the exponent is determined by the ratio of the energy gaps. This analysis can be repeated for temperature dependences of the variable range hopping (VRH) type, exp[−(*T*_0_/*T*)]*^a^*, and the same result is obtained, with (∆*E*_H_/∆*E_x_*) modified to (*T*_0H_/*T*_0_*_x_*)*^a^*.

There are two caveats to this result. First, we have neglected any temperature-dependent prefactors in the variable range hopping. Prefactors are notoriously difficult to determine experimentally; the reason being that the effect of the prefactor only becomes significant at higher temperatures, whereas in practice VRH is washed out at high temperatures by thermal activation. Second, the exponent *a* in the VRH *T* dependence was assumed to be the same for *R_x_* and ∆*R*_H_.

Experimentally, quite often a linear relationship has been observed between *R_x_* and ∆*R*_H_ [10–13,16–18], implying that *R_x_* and ∆*R*_H_ experience the same ∆*E* or *T*_0_. Isolated cases have shown deviations from this linear form [13, 17], although it was not stated in those cases whether the data fit a power law dependence. Mandal and Ravishankar [29] have applied the self-consistent Born approximation to calculate the effect of temperature and impurities on the Hall and longitudinal conductivities. They found that for a certain range of temperatures and level broadening *R_x_* was proportional to −∆*R*_H_, as seen in the experiments.

## 5. Energy Gap and Effective *g*-Factor

We might expect the energy gaps to be comparable to the cyclotron energy, *E*_c_ =*ħω*_c_ =*ħeB*/m*, where the effective mass m* is 6.8 % of the free electron mass *m*_e_ in GaAs. Instead, we see from Table 1 that the energy gaps range from about 2/3 (*i* = 2) to 1/3 (*i* = 8) of the cyclotron energy. One possible explanation involves the spin splitting. Since the Landau levels are split by the Zeeman energy, the energy gap between the filled higher energy spin-split level (even filling factors) and the next low energy spin-split Landau level would be reduced from the pure cyclotron energy. This argument would work well for *i* = 2. However, it is unclear how to extend this argument to the higher filling factors, since the spin-splitting is considerably smaller there, yet the energy gap is also, proportionally, smaller than for *i* = 2.

This problem has been discussed in the literature. Several solutions have been proposed, including the effect of a finite thickness of the electron layer [30], and disorder broadening of the Landau levels due to impurities in the bulk [31, 32]. We apply an elementary discussion of the latter effect to the results found for GaAs(7).

The effect of charged impurities in the bulk is to broaden the Landau levels, giving them a finite bandwidth. This finite width would reduce the effective energy gap between neighboring levels. In the simplest case, we can assume the effect of this Landau level broadening is to reduce the energy gap by a constant amount, *Γ*. If we consider the energy gap otherwise to be composed of the cyclotron energy and the Zeeman energy, the expression for the energy gap, ∆*E*, becomes

$$\Delta E=\hbar \omega_{c}-g\mu_{B}B-\Gamma$$ (7)

where *g* is the effective *g*-factor. Equation (7) can be rearranged to show the linearity in *B*,

$$\Delta E=\frac{\hbar e}{m^{*}}\left(1-\frac{g}{2}\frac{m^{*}}{m_{e}}\right)B-\Gamma .$$ (8)

Thus, by applying a linear fit to the energy gap as a function of magnetic field, we can deduce the offset *Γ*, as well as the *g*-factor.

**Fig. 8:**
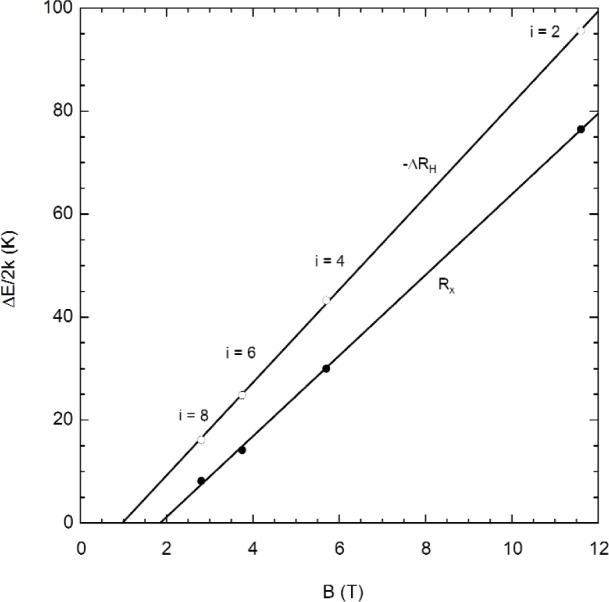
Activation energy gap in temperature units as a function of magnetic field for - *R*_H_ (open circles) and *R_x_* (closed circles). The solid lines are linear least squares fits.

Such a fit is shown in Fig. 8. For *R_x_* the offset obtained is given by Γ/2*k* = 14.5 K. If this offset is then added to the measured energy gaps, corrected energy gaps for all filling factors are about 78% *ħω*_c_, which in turn implies a *g*-factor of about 6.5 in Eq. (7). For −∆*R*_H_, the offset obtained is given by Γ/2*k* = 8.76 K. If this offset is then added to the measured energy gaps, all corrected gaps are about 90% *ħω*_c_, which in turn implies a *g*-factor of about 3.0, about half of the value found for *R_x_*.

From Eq. (8) we can also deduce the magnetic field below which there is no energy gap. For *R_x_* this corresponds to

$$B_{0}=\frac{\Gamma /2k}{slope}=\frac{14.46}{7.83}=1.85T$$ (9)

which is equivalent to a filling factor *i* ≈ 12. As described in Ref. [27], the longitudinal conductivity *σ_xx_*(*B*,*T*) in GaAs(7) is described well by a perturbative treatment for low-magnetic field [see Ref. (34)]. The Schubnikov-deHaas oscillations obey the following relation, for *B* = 2 *T*:

$$\begin{array}{c} \sigma_{xx}=\left(B,T\right)=F^{\left(0\right)}\left(B\right)-F^{\left(1\right)}\left(B\right)\frac{2\pi^{2}kT}{\hbar \omega_{c}}\\ \csc h\left(\frac{2\pi^{2}kT}{\hbar \omega_{c}}\right)\cos\left(\frac{2\pi E_{F}}{\hbar \omega_{c}}\right). \end{array}$$ (10)

Here *E*_F_ is the Fermi energy, and *F*^(0)^ and *F*^(1^) are slowly-varying functions, unspecified by the theory. The longitudinal conductivity *σ*_xx_ is related to the resistivities by $\sigma_{\text{xx}}=\rho_{xx}/\left({\rho_{xx}}^{2}+{\rho_{xx}}^{2}\right)$.

The Ando low-*B* theory [33] is expected to hold when *ħω*_c_≪*E_F_*. The fact that the theory works only below about 2 K is consistent with Eq. (9), which predicts a small energy gap over the same range in *B*. A similar calculation for ∆*R*_H_ yields a minimum field of 0.97 *T*, or a filling factor of about 24.

Why ∆*R*_H_ and *R_x_* energy gaps, offsets, and *g*-factors differ is not altogether clear. The difference in the offset, *Γ*, may be due to inhomogeneities in the device. Since *R*_H_ and *R_x_* are measured over different regions of the device, a non-uniform concentration of impurities in the bulk could lead to different broadening of the Landau levels.

The value of *Γ* in the Sasaki-Ezawa model [32] is related to the average distance of the impurities in the bulk from the 2DEG. In the *R_x_* case, *Γ* = 2*k ×* 14.5 K = 2.5 meV, which implies the impurities are about 45 nm from the 2D layer. In the *R*_H_ case *Γ* = 1.5 meV, which implies the impurities are about 70 nm from the 2D electron layer. These distances are both consistent with the device growth parameters (see Ref. [27]). If, for some reason, the average distance of the impurities from the 2DEG was larger on the side of the device close to the Hall voltage than on the side of the device close to ground, then the average distance of the impurities from the 2DEG would be greater for Hall measurements than for *R_x_* measurements (which are always made close to ground). One way to investigate this would be to make temperature dependence measurements of *R_x_* on the off-ground side of the device. If the resultant *Γ* is closer to 1.5 meV than 2.5 meV, this would support the above conjecture.

The reason why the effective *g*-factor is different for *R*_H_ and *R_x_* is less clear. Although, given that the calculation of the *g*-factor enhancement by Ando and Uemura [30] made use of impurity broadening of the Landau levels, and the *g*-factor enhancement is observed experimentally to be sample dependent, it seems quite possible that the inhomogeneities could also be responsible for the variation in the *g*-factor.

Finally, we show that the Sazaki-Ezawa model yields the *δ*(*i*) values in Table 2, using the fitted values of *g* and *Γ*. To see this, we recall from Eq. (6) that *δ* = ∆*E*_H_/∆*E_x_*, and replacing ∆*E*_H_ and ∆*E_x_* using Eq. (7), we arrive at the following expression for *δ*:

$$\delta =\frac{1-g_{H}\left(m^{*}/2m_{e}\right)-i\left(\Gamma_{H}/\hbar \omega_{1}\right)}{1-g_{x}\left(m^{*}/2m_{e}\right)-i\left(\Gamma_{x}/\hbar \omega_{1}\right)}.$$ (11)

Here, *ω*_1_ = *eB*_1_/m^*^ is the cyclotron frequency at *i* = 1, and in our experiments *B*_1_ = 23.0 *T*, hence *ħω*_c_ = 39.2meV. Using the numerical values for *g* and *Γ*, the numerical value of *δ* is given by:

$$\delta \left(i\right)=\frac{0.898-0.038i}{0.779-0.064i}.$$ (12)

Substituting *i* = 2, 4, 6, and 8, we find *δ* = 1.26, 1.43, 1.70, and 2.22, respectively. This is consistent with the values found experimentally (see Table 2): *δ* = 1.25, 1.44, 1.75, and 1.97. The largest discrepancy is for *i* = 8, which is not surprising, since from Eq. (7) we can see that for large *i* (or small *B*) the level broadening *Γ* is comparable to the level spacing, and the model is no longer valid. For small *i*, we can Taylor expand the denominator of Eq. (11), yielding the approximate expression:

$$\delta =1+\Delta g\left(m^{*}/2m_{e}\right)+i\left(\Delta \Gamma /\hbar \omega_{1}\right)$$ (13)

where ∆*g = g_x_ − g*_H_, and ∆ *Γ = Γ _x_* − *Γ*_H_.

## 6. Conclusion

By examining the temperature dependence of *R_x_* we found two distinct regimes. Between 4.2 K and 10 K we observed the expected activated behavior, due to an energy gap. By using a simple model of impurity broadening of the energy levels, we found the gap to be about 78% *ħω*_c_. The difference is due to the enhanced spin-splitting, for which we obtained an effective *g*-factor of 6.5. This is comparable to values quoted in the literature [34–38].

In addition, by examining ∆*R*_H_(*T*) ≡ *R*_H_(*T*) − *R*_H_(0) as a function of *R_x_*(*T*), we were able to determine that ∆*R*_H_(*T*) is also activated, with an energy gap of about 90% *ħω*_c_, implying an effective *g*-factor of 3.0.

At lower temperatures (1.4 K to 4 K) *R_x_* for all four filling factors clearly exhibited a power-law dependence on temperature, with powers ranging from 2.5 for *i* = 8 to 10.9 for *i* = 2. However, *i* = 2 showed a power law dependence over a higher temperature range than the other filling factors (4 K to 7 K). At lower temperatures the *R_x_*(*T*) curve flattened out for *i* = 2. In no case were we able to fit temperature dependences predicted by the theory of variable range hopping, which is typically observed in these kind of transport experiments (although it is almost always measured away from the *R_x_* minima to gain enough sensitivity).

Finally, we note a result obtained that may have the most practical impact for resistance metrology, which is the power law relationship between ∆*R*_H_(*T*) and *R_x_*(*T*). We found a power law to hold for all three *R*_H_ probe sets, and all four filling factors, over the entire temperature range measured. While we would expect power law behavior, or even a linear relationship, if ∆*R*_H_(*T*) showed similar temperature dependence as *R_x_*(*T*), it is not clear why *R_x_* and ∆*R*_H_ follow the same power law over the entire temperature range. However, in practice this can be very useful, as it gives an empirical tool for establishing the limiting value of *R*_H_ as *R_x_* →0. We found the powers, which range from about 1.25 for *i* = 2 to about 2.00 for *i* = 8, could be explained satisfactorily by applying the Sazaki-Ezawa model of bulk impurities and spin-splitting.

## Figures and Tables

**Fig. 7a, b:**
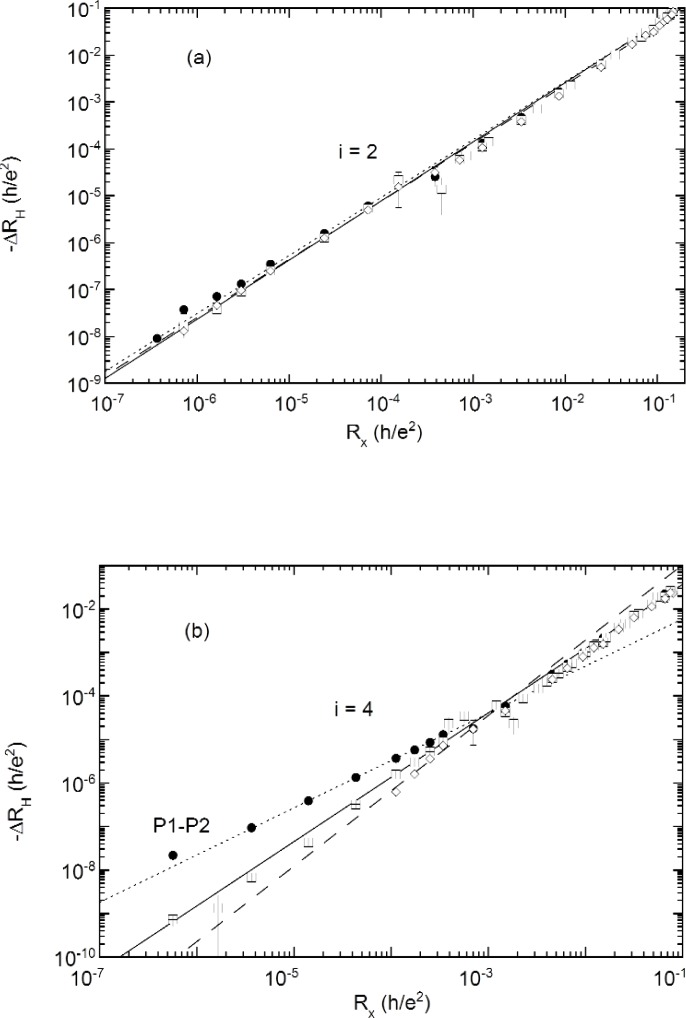
Parametric plot of −∆*R*_H_ vs *R_x_* as the temperature is varied for (a) *i* = 2, (b) *i* = 4. Three different probe sets (Fig. 1) were used to measure *R*_H_: P1–P2 (solid circles), P3–P4 (open squares), and P5–P6 (open diamonds). Note that the power law dependence holds over many orders of magnitude in ∆*R*_H_ and *R_x_*, e.g., *seven* orders of magnitude for *i* = 2.

**Fig. 7c, d:**
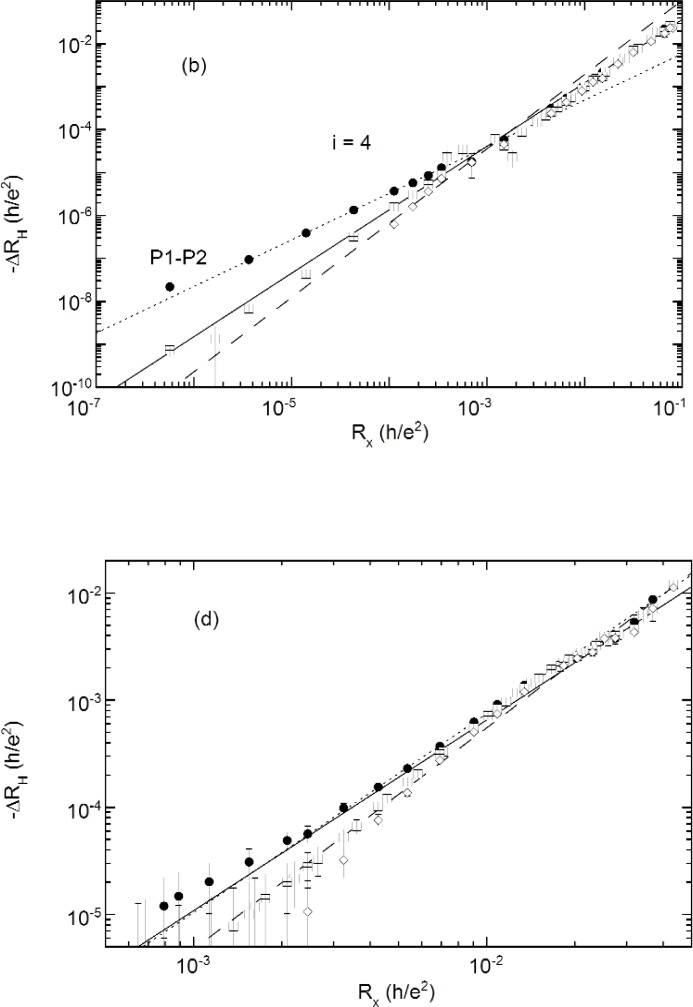
Parametric plot of −∆*R*_H_ vs *R_x_* as the temperature is varied for (c) *i* = 6, (d) *i* = 8. Three different probe sets (Fig. 1) were used to measure *R*_H_: P1–P2 (solid circles), P3–P4 (open squares), and P5–P6 (open diamonds). Note that the power law dependence holds over many orders of magnitude in ∆*R*_H_ and *R_x_*, e.g., *seven* orders of magnitude for *i* = 2.

**Table 1:** Parameters of the least squares fits to *R_x_* (*T*) shown in Fig. 5. ∆*E* and *E*_c_ are defined in Sec. 5

	Thermal activation			Power law		Empirical model		
	*R_x_*_0_(*h/e*^2^)	*T*_0_(K)	∆*E*/*E*_c_	*a*(*h*/*e*^2^K^γ^)	*γ*	*R_x_*_2_(*h/e*^2^)	*T*_2_(*K*)	*α*
*i* = 2	4.68	76.5	0.66	4 × 10^−14^	10.9	1.3 × 10^−9^	1.33	1.50
*i* = 4	0.38	30.0	0.53	5 × 10^−8^	6.1			
*i* = 6	0.11	14.2	0.38	2 × 10^−5^	3.6			
*i* = 8	0.06	8.2	0.29	3 × 10^−4^	2.5			

**Table 2:** Parameters of least squares fits to −∆*R*_H_(*T*) = *sRx* (*T*)^δ^, as shown in Fig. 7. All four even integer filling factors are shown, with three different *R*_H_ probe sets and the average over all three probe sets for each filling factor. There is an uncertainty of one unit in the last digit associated with the values in this table

	*R*_H_ Probe Set	*s*	δ
*i* = 2	P1–P2	0.81	1.24
*i* = 2	P3–P4	0.87	1.26
*i* = 2	P5–P6	0.71	1.24
***i* = 2**	average	**0.82**	**1.25**
*i* = 4	P1–P2	0.07	1.09
*i* = 4	P3–P4	1.12	1.48
*i* = 4	P5–P6	5.64	1.73
***i* = 4**	average	**0.93**	**1.44**
*i* = 6	P1–P2	1.9	1.66
*i* = 6	P3–P4	3.4	1.80
*i* = 6	P5–P6	3.4	1.82
***i* = 6**	average	**2.6**	**1.75**
*i* = 8	P1–P2	3.8	1.85
*i* = 8	P3–P4	2.4	1.78
*i* = 8	P5–P6	7.5	2.07
***i* = 8**	average	**5.5**	**1.97**

## Footnotes

1 Certain commercial equipment, instruments, or materials are identified in this paper to foster understanding. Such identification does not imply recommendation or endorsement by the National Institute of Standards and Technology, nor does it imply that the materials or equipment identified are necessarily the best available for the purpose.
